# THE CONCISE GUIDE TO PHARMACOLOGY 2017/18: Nuclear hormone receptors

**DOI:** 10.1111/bph.13880

**Published:** 2017-10-21

**Authors:** Stephen PH Alexander, John A Cidlowski, Eamonn Kelly, Neil V Marrion, John A Peters, Elena Faccenda, Simon D Harding, Adam J Pawson, Joanna L Sharman, Christopher Southan, Jamie A Davies, CGTP Collaborators

**Affiliations:** ^1^ School of Life Sciences Nottingham NG7 2UH UK; ^2^ National Institute of Environmental Health Sciences, National Institutes of Health Department of Health and Human Services Research Triangle Park NC 27709 USA; ^3^ School of Physiology, Pharmacology and Neuroscience University of Bristol Bristol BS8 1TD UK; ^4^ Neuroscience Division, Medical Education Institute, Ninewells Hospital and Medical School University of Dundee Dundee DD1 9SY UK; ^5^ Centre for Integrative Physiology University of Edinburgh Edinburgh EH8 9XD UK

## Abstract

The Concise Guide to PHARMACOLOGY 2017/18 provides concise overviews of the key properties of nearly 1800 human drug targets with an emphasis on selective pharmacology (where available), plus links to an open access knowledgebase of drug targets and their ligands (www.guidetopharmacology.org), which provides more detailed views of target and ligand properties. Although the Concise Guide represents approximately 400 pages, the material presented is substantially reduced compared to information and links presented on the website. It provides a permanent, citable, point‐in‐time record that will survive database updates. The full contents of this section can be found at http://onlinelibrary.wiley.com/doi/10.1111/bph.13880/full. Nuclear hormone receptors are one of the eight major pharmacological targets into which the Guide is divided, with the others being: G protein‐coupled receptors, ligand‐gated ion channels, voltage‐gated ion channels, other ion channels, catalytic receptors, enzymes and transporters. These are presented with nomenclature guidance and summary information on the best available pharmacological tools, alongside key references and suggestions for further reading. The landscape format of the Concise Guide is designed to facilitate comparison of related targets from material contemporary to mid‐2017, and supersedes data presented in the 2015/16 and 2013/14 Concise Guides and previous Guides to Receptors and Channels. It is produced in close conjunction with the Nomenclature Committee of the Union of Basic and Clinical Pharmacology (NC‐IUPHAR), therefore, providing official IUPHAR classification and nomenclature for human drug targets, where appropriate. © 2015 The British Pharmacological Society

## Conflict of interest

The authors state that there are no conflicts of interest to declare.

© 2017 The Authors. British Journal of Pharmacology published by John Wiley & Sons Ltd on behalf of British Pharmacological Society. This is an open access article under the terms of the Creative Commons Attribution License, which permits use, distribution and reproduction in any medium, provided the original work is properly cited.

## Overview

Nuclear receptors are specialised transcription factors with commonalities of sequence and structure, which bind as homo‐ or heterodimers to specific consensus sequences of DNA (response elements) in the promoter region of particular target genes. They regulate (either promoting or repressing) transcription of these target genes in response to a variety of endogenous ligands. Endogenous agonists are hydrophobic entities which, when bound to the receptor promote conformational changes in the receptor to allow recruitment (or dissociation) of protein partners, generating a large multiprotein complex.

Two major subclasses of nuclear receptors with identified endogenous agonists can be identified: steroid and non‐steroid hormone receptors. Steroid hormone receptors function typically as dimeric entities and are thought to be resident outside the nucleus in the unliganded state in a complex with chaperone proteins, which are liberated upon agonist binding. Migration to the nucleus and interaction with other regulators of gene transcription, including RNA polymerase, acetyltransferases and deacetylases, allows gene transcription to be regulated. Non‐steroid hormone receptors typically exhibit a greater distribution in the nucleus in the unliganded state and interact with other nuclear receptors to form heterodimers, as well as with other regulators of gene transcription, leading to changes in gene transcription upon agonist binding.

Selectivity of gene regulation is brought about through interaction of nuclear receptors with particular consensus sequences of DNA, which are arranged typically as repeats or inverted palindromes to allow accumulation of multiple transcription factors in the promoter regions of genes.

## Family structure

S209 1A. Thyroid hormone receptors S210 1B. Retinoic acid receptors S210 1C. Peroxisome proliferator‐activated receptors S211 1D. Rev‐Erb receptors S212 1F. Retinoic acid‐related orphans S212 1H. Liver X receptor‐like receptors S213 1I. Vitamin D receptor‐like receptors S214 2A. Hepatocyte nuclear factor‐4 receptors S215 2B. Retinoid X receptors S216 2C. Testicular receptors S216 2E. Tailless‐like receptors S217 2F. COUP‐TF‐like receptors S217 3B. Estrogen‐related receptors S218 4A. Nerve growth factor IB‐like receptors S219 5A. Fushi tarazu F1‐like receptors S219 6A. Germ cell nuclear factor receptors S220 0B. DAX‐like receptors S221 Steroid hormone receptors S221 3A. Estrogen receptors S221 3C. 3‐Ketosteroid receptors

## 1A. Thyroid hormone receptors

### Overview

Thyroid hormone receptors (TRs, nomenclature as agreed by the NC‐IUPHAR Subcommittee on Nuclear Hormone Receptors [41]) are nuclear hormone receptors of the NR1A family, with diverse roles regulating macronutrient metabolism, cognition and cardiovascular homeostasis. TRs are activated by thyroxine (T_4_) and thyroid hormone (triiodothyronine). Once activated by a ligand, the receptor acts as a transcription factor either as a monomer, homodimer or heterodimer with members of the retinoid X receptor family. NH‐3 has been described as an antagonist at TRs with modest selectivity for TR*β*[110].

**Table:** 

Nomenclature	Thyroid hormone receptor‐*α*	Thyroid hormone receptor‐*β*
Systematic nomenclature	NR1A1	NR1A2
HGNC, UniProt	THRA, P10827	THRB, P10828
Rank order of potency	triiodothyronine>T_4_	triiodothyronine>T_4_
Agonists	dextrothyroxine [19]	dextrothyroxine [19]
Selective agonists	–	sobetirome [26, 130]

### Comments

An interaction with integrin *α*V*β*3 has been suggested to underlie plasma membrane localization of TRs and non‐genomic signalling [8].One splice variant, TR*α*_2_, lacks a functional DNA‐binding domain and appears to act as a transcription suppressor.

Although radioligand binding assays have been described for these receptors, the radioligands are not commercially available.

### Further reading on 1A. Thyroid hormone receptors

Davis PJ et al. (2016) Nongenomic actions of thyroid hormone. Nat Rev Endocrinol 12: 111‐21 [PMID:26668118]

Elbers LP et al. (2016) Thyroid Hormone Mimetics: the Past, Current Status and Future Challenges. Curr Atheroscler Rep 18: 14 [PMID:26886134]

Flamant F et al. (2006) International Union of Pharmacology. LIX. The pharmacology and classification of the nuclear receptor superfamily: thyroid hormone receptors. Pharmacol. Rev. 58: 705‐11 [PMID:17132849]

Mendoza A et al. (2017) New insights into thyroid hormone action. Pharmacol Ther 173: 135‐145 [PMID:28174093]

## 1B. Retinoic acid receptors

### Overview

Retinoic acid receptors (nomenclature as agreed by the NC‐IUPHAR Subcommittee on Nuclear Hormone Receptors [46]) are nuclear hormone receptors of the NR1B family activated by the vitamin A‐derived agonists tretinoin (ATRA) and alitretinoin, and the RAR‐selective synthetic agonists TTNPB and adapalene. BMS493 is a family‐selective antagonist [47].

**Table:** 

Nomenclature	Retinoic acid receptor‐*α*	Retinoic acid receptor‐*β*	Retinoic acid receptor‐*γ*
Systematic nomenclature	NR1B1	NR1B2	NR1B3
HGNC, UniProt	RARA, P10276	RARB, P10826	RARG, P13631
Agonists	tretinoin [25]	tretinoin [25]	tretinoin [25]
Sub/family‐selective agonists	tazarotene [25]	tazarotene [25], adapalene [24]	tazarotene [25], adapalene [24]
Selective agonists	BMS753 [53], tamibarotene [146], Ro 40‐6055 [33]	AC261066 [89], AC55649 [88, 89]	AHPN [24]
Selective antagonists	Ro 41‐5253 (pIC_50_ 6.3–7.2) [2, 69]	–	MM 11253 [76]

### Comments

Ro 41‐5253 has been suggested to be a PPAR*γ* agonist [129]. LE135 is an antagonist with selectivity for RAR*α* and RAR*β* compared with RAR*γ*[84].

### Further reading on 1B. Retinoic acid receptors

Duong V et al. (2011) The molecular physiology of nuclear retinoic acid receptors. From health to disease. Biochim. Biophys. Acta 1812: 1023‐31 [PMID:20970498]

Germain P et al. (2006) International Union of Pharmacology. LX. Retinoic acid receptors. Pharmacol. Rev. 58: 712‐25 [PMID:17132850]

Larange A et al. (2016) Retinoic Acid and Retinoic Acid Receptors as Pleiotropic Modulators of the Immune System. Annu Rev Immunol 34: 369‐94 [PMID:27168242]

Saeed A et al. (2017) The interrelationship between bile acid and vitamin A homeostasis. Biochim Biophys Acta 1862: 496‐512 [PMID:28111285]

## 1C. Peroxisome proliferator‐activated receptors

### Overview

Peroxisome proliferator‐activated receptors (PPARs, nomenclature as agreed by the NC‐IUPHAR Subcommittee on Nuclear Hormone Receptors [101]) are nuclear hormone receptors of the NR1C family, with diverse roles regulating lipid homeostasis, cellular differentiation, proliferation and the immune response. PPARs have many potential endogenous agonists [13, 101], including 15‐deoxy‐Δ^12,14^‐PGJ_2_, prostacyclin (PGI_2_), many fatty acids and their oxidation products, lysophosphatidic acid (LPA) [98], 13‐HODE, 15S‐HETE, Paz‐PC, azelaoyl‐PAF and leukotriene B4 (LTB_4_). Bezafibrate acts as a non‐selective agonist for the PPAR family [155]. These receptors also bind hypolipidaemic drugs (PPAR*α*) and anti‐diabetic thiazolidinediones (PPAR*γ*), as well as many non‐steroidal anti‐inflammatory drugs, such as sulindac and indomethacin. Once activated by a ligand, the receptor forms a heterodimer with members of the retinoid X receptor family and can act as a transcription factor. Although radioligand binding assays have been described for all three receptors, the radioligands are not commercially available. Commonly, receptor occupancy studies are conducted using fluorescent ligands and truncated forms of the receptor limited to the ligand binding domain.

**Table:** 

Nomenclature	Peroxisome proliferator‐activated receptor‐*α*	Peroxisome proliferator‐activated receptor‐*β*/*δ*	Peroxisome proliferator‐activated receptor‐*γ*
Systematic nomenclature	NR1C1	NR1C2	NR1C3
HGNC, UniProt	PPARA, Q07869	PPARD, Q03181	PPARG, P37231
Selective agonists	GW7647 [17, 18], CP‐775146 [67], pirinixic acid [155], gemfibrozil [31]	GW0742X [50, 148], GW501516 [112]	GW1929 [17], bardoxolone (Partial agonist) [149], rosiglitazone [59, 80, 86, 161], troglitazone [59, 161], pioglitazone [7, 59, 127, 161], ciglitazone [59]
Selective antagonists	GW6471 (pIC_50_ 6.6) [158]	GSK0660 (pIC_50_ 6.5) [131]	T0070907 (p*K*_i_ 9) [77], GW9662 (Irreversible inhibition) (pIC_50_ 8.1) [78], CDDO‐Me (p*K*_i_ 6.9) [149]

### Comments

As with the estrogen receptor antagonists, many agents show tissue‐selective efficacy (e.g. [12, 109, 124]). Agonists with mixed activity at PPAR*α* and PPAR*γ* have also been described (e.g [35, 52, 159]).

### Further reading on 1C. Peroxisome proliferator‐activated receptors

Cheang WS et al. (2015) The peroxisome proliferator‐activated receptors in cardiovascular diseases: experimental benefits and clinical challenges. Br J Pharmacol 172: 5512‐22 [PMID:25438608]

Gross B et al. (2017) PPARs in obesity‐induced T2DM, dyslipidaemia and NAFLD. Nat Rev Endocrinol 13: 36‐49 [PMID:27636730]

Hallenborg P et al. (2016) The elusive endogenous adipogenic PPARgamma agonists: Lining up the suspects. Prog Lipid Res 61: 149‐62 [PMID:26703188]

Michalik L et al. (2006) International Union of Pharmacology. LXI. Peroxisome proliferator‐activated receptors. Pharmacol. Rev. 58: 726‐41 [PMID:17132851]

Sauer S. (2015) Ligands for the Nuclear Peroxisome Proliferator‐Activated Receptor Gamma. Trends Pharmacol Sci 36: 688‐704 [PMID:26435213]

## 1D. Rev‐Erb receptors

### Overview

Rev‐erb receptors (nomenclature as agreed by the NC‐IUPHAR Subcommittee on Nuclear Hormone Receptors [6]) have yet to be officially paired with an endogenous ligand, but are thought to be activated by heme.

**Table:** 

Nomenclature	Rev‐Erb‐*α*	Rev‐Erb‐*β*
Systematic nomenclature	NR1D1	NR1D2
HGNC, UniProt	NR1D1, P20393	NR1D2, Q14995
Endogenous agonists	heme [121, 160]	heme [97, 121, 160]
Selective agonists	GSK4112 [51], GSK4112 [70]	–
Selective antagonists	SR8278 (pIC_50_ 6.5) [70]	–

### Further reading on 1D. Rev‐Erb receptors

Benoit G et al. (2006) International Union of Pharmacology. LXVI. Orphan nuclear receptors. Pharmacol. Rev. 58: 798‐836 [PMID:17132856]

Gonzalez‐Sanchez E et al. (2015) Nuclear receptors in acute and chronic cholestasis. Dig Dis 33: 357‐66 [PMID:26045270]

Gustafson CL et al. (2015) Emerging models for the molecular basis of mammalian circadian timing. Biochemistry 54: 134‐49 [PMID:25303119]

Sousa EH et al. (2017) Drug discovery targeting heme‐based sensors and their coupled activities. J Inorg Biochem 167: 12‐20 [PMID:27893989]

## 1F. Retinoic acid‐related orphans

### Overview

Retinoic acid receptor‐related orphan receptors (ROR, nomenclature as agreed by the NC‐IUPHAR Subcommittee on Nuclear Hormone Receptors [6]) have yet to be assigned a definitive endogenous ligand, although ROR*α* may be synthesized with a ‘captured’ agonist such as cholesterol[65, 66].

**Table:** 

Nomenclature	RAR‐related orphan receptor‐*α*	RAR‐related orphan receptor‐*β*	RAR‐related orphan receptor‐*γ*
Systematic nomenclature	NR1F1	NR1F2	NR1F3
HGNC, UniProt	RORA, P35398	RORB, Q92753	RORC, P51449
Endogenous agonists	cholesterol [66, 114]	–	–
Selective agonists	7‐hydroxycholesterol [14], cholesterol sulphate [14, 66]	–	–

### Comments

tretinoin shows selectivity for ROR*β* within the ROR family [136]. ROR*α* has been suggested to be a nuclear receptor responding to melatonin[154].

### Further reading on 1F. Retinoic acid‐related orphans

Benoit G et al. (2006) International Union of Pharmacology. LXVI. Orphan nuclear receptors. Pharmacol. Rev. 58: 798‐836 [PMID:17132856]

Cyr P et al. (2016) Recent progress on nuclear receptor RORgamma modulators. Bioorg Med Chem Lett 26: 4387‐93 [PMID:27542308]

Dahlman‐Wright K et al. (2006) Overview of nomenclature of nuclear receptors. Pharmacol Rev 58: 685‐704 [PMID:17132848]

Guillemot‐Legris O et al. (2016) Oxysterols in Metabolic Syndrome: From Bystander Molecules to Bioactive Lipids. Trends Mol Med 22: 594‐614 [PMID:27286741]

Mutemberezi V et al. (2016) Oxysterols: From cholesterol metabolites to key mediators. Prog Lipid Res 64: 152‐169 [PMID:27687912]

## 1H. Liver X receptor‐like receptors

### Overview

Liver X and farnesoid X receptors (LXR and FXR, nomenclature as agreed by the NC‐IUPHAR Subcommittee on Nuclear Hormone Receptors [105]) are members of a steroid analogue‐activated nuclear receptor subfamily, which form heterodimers with members of the retinoid X receptor family. Endogenous ligands for LXRs include hydroxycholesterols (OHC), while FXRs appear to be activated by bile acids.

**Table:** 

Nomenclature	Farnesoid X receptor	Farnesoid X receptor‐*β*	Liver X receptor‐*α*	Liver X receptor‐*β*
Systematic nomenclature	NR1H4	NR1H5	NR1H3	NR1H2
HGNC, UniProt	NR1H4, Q96RI1	NR1H5P, –	NR1H3, Q13133	NR1H2, P55055
Potency order	chenodeoxycholic acid>lithocholic acid, deoxycholic acid [92, 115]	–	20S‐hydroxycholesterol, 22R‐hydroxycholesterol, 24(S)‐hydroxycholesterol>25‐hydroxycholesterol, 27‐hydroxycholesterol [79]	20S‐hydroxycholesterol, 22R‐hydroxycholesterol, 24(S)‐hydroxycholesterol>25‐hydroxycholesterol, 27‐hydroxycholesterol [79]
Endogenous agonists	–	lanosterol [113] – Mouse	–	–
Selective agonists	GW4064 [94], obeticholic acid [116], fexaramine [36]	–	–	–
Selective antagonists	guggulsterone (pIC_50_ 5.7–6) [157]	–	–	–

### Comments

T0901317[122] and GW3965[27] are synthetic agonists acting at both LXR*α* and LXR*β* with less than 10‐fold selectivity.

### Further reading on 1H. Liver X receptor‐like receptors

Courtney R et al. (2016) LXR Regulation of Brain Cholesterol: From Development to Disease. Trends Endocrinol Metab 27: 404‐14 [PMID:27113081]

Gadaleta RM et al. (2017) Bile acids and colon cancer: Is FXR the solution of the conundrum? Mol Aspects Med [PMID:28400119]

Merlen G et al. (2017) Bile acids and their receptors during liver regeneration: "Dangerous protectors". Mol Aspects Med [PMID:28302491]

Moore DD et al. (2006) International Union of Pharmacology. LXII. The NR1H and NR1I receptors: constitutive androstane receptor, pregnene X receptor, farnesoid X receptor alpha, farnesoid X receptor beta, liver X receptor alpha, liver X receptor beta, and vitamin D receptor. Pharmacol Rev 58: 742‐59 [PMID:17132852]

Mouzat K et al. (2016) Liver X receptors: from cholesterol regulation to neuroprotection‐a new barrier against neurodegeneration in amyotrophic lateral sclerosis? Cell Mol Life Sci 73: 3801‐8

Schulman IG. (2017) Liver X receptors link lipid metabolism and inflammation. FEBS Lett [PMID:28555747]

## 1I. Vitamin D receptor‐like receptors

### Overview

Vitamin D (VDR), Pregnane X (PXR) and Constitutive Androstane (CAR) receptors (nomenclature as agreed by the NC‐IUPHAR Subcommittee on Nuclear Hormone Receptors [105]) are members of the NR1I family of nuclear receptors, which form heterodimers with members of the retinoid X receptor family. PXR and CAR are activated by a range of exogenous compounds, with no established endogenous physiological agonists, although high concentrations of bile acids and bile pigments activate PXR and CAR [105].

**Table:** 

Nomenclature	Vitamin D receptor	Pregnane X receptor	Constitutive androstane receptor
Systematic nomenclature	NR1I1	NR1I2	NR1I3
HGNC, UniProt	VDR, P11473	NR1I2, O75469	NR1I3, Q14994
Endogenous agonists	1,25‐dihydroxyvitamin D3 [11, 39]	17*β*‐estradiol [64]	–
Selective agonists	seocalcitol [28, 153], doxercalciferol	hyperforin [106, 152], 5*β*‐pregnane‐3,20‐dione [64], lovastatin [81], rifampicin [15, 81]	TCPOBOP [144] – Mouse, CITCO [91]
Selective antagonists	TEI‐9647 (pIC_50_ 8.2) [126] – Chicken, ZK159222 (pIC_50_ 7.5) [42, 60]	–	–
Comments	–	–	clotrimazole [107] and T0901317 [68] although acting at other sites, function as antagonists of the constitutive androstane receptor.

### Further reading on 1I. Vitamin D receptor‐like receptors

Benoit G et al. (2006) International Union of Pharmacology. LXVI. Orphan nuclear receptors. Pharmacol. Rev. 58: 798‐836 [PMID:17132856]

Long MD et al. (2015) Vitamin D receptor and RXR in the post‐genomic era. J Cell Physiol. 230: 758‐66 [PMID:25335912]

Moore DD et al. (2006) International Union of Pharmacology. LXII. The NR1H and NR1I receptors: constitutive androstane receptor, pregnene X receptor, farnesoid X receptor alpha, farnesoid X receptor beta, liver X receptor alpha, liver X receptor beta, and vitamin D receptor. Pharmacol. Rev. 58: 742‐59 [PMID:17132852]

## 2A. Hepatocyte nuclear factor‐4 receptors

### Overview

The nomenclature of hepatocyte nuclear factor‐4 receptors is agreed by the NC‐IUPHAR Subcommittee on Nuclear Hormone Receptors [6]. While linoleic acid has been identified as the endogenous ligand for HNF4*α* its function remains ambiguous [163]. HNF4*γ* has yet to be paired with an endogenous ligand.

**Table:** 

Nomenclature	Hepatocyte nuclear factor‐4‐*α*	Hepatocyte nuclear factor‐4‐*γ*
Systematic nomenclature	NR2A1	NR2A2
HGNC, UniProt	HNF4A, P41235	HNF4G, Q14541
Endogenous agonists	linoleic acid [163]	–
Selective antagonists	BI6015 [71]	–
Comments	HNF4*α* has constitutive transactivation activity [163] and binds DNA as a homodimer [63].	–

### Further reading on 2A. Hepatocyte nuclear factor‐4 receptors

Benoit G et al. (2006) International Union of Pharmacology. LXVI. Orphan nuclear receptors. Pharmacol. Rev. 58: 798‐836 [PMID:17132856]

Germain P et al. (2006) Overview of nomenclature of nuclear receptors. Pharmacol. Rev. 58: 685‐704 [PMID:17132848]

Garattini E et al. (2016) Lipid‐sensors, enigmatic‐orphan and orphan nuclear receptors as therapeutic targets in breast‐cancer. Oncotarget 7: 42661‐42682 [PMID:26894976]

Lu H. (2016) Crosstalk of HNF4alpha with extracellular and intracellular signaling pathways in the regulation of hepatic metabolism of drugs and lipids. Acta Pharm Sin B 6: 393‐408 [PMID:27709008]

Walesky CE et al. (2015) Role of hepatocyte nuclear factor 4alpha (HNF4alpha) in cell proliferation and cancer. Gene Expr 16: 101‐8[PMID:25700366]

## 2B. Retinoid X receptors

### Overview

Retinoid X receptors (nomenclature as agreed by the NC‐IUPHAR Subcommittee on Nuclear Hormone Receptors [45]) are NR2B family members activated by alitretinoin and the RXR‐selective agonists bexarotene and LG100268, sometimes referred to as rexinoids. UVI3003[108] and HX 531 [37] have been described as a pan‐RXR antagonists. These receptors form RXR‐RAR heterodimers and RXR‐RXR homodimers [22, 96].

**Table:** 

Nomenclature	Retinoid X receptor‐*α*	Retinoid X receptor‐*β*	Retinoid X receptor‐*γ*
Systematic nomenclature	NR2B1	NR2B2	NR2B3
HGNC, UniProt	RXRA, P19793	RXRB, P28702	RXRG, P48443
Sub/family‐selective agonists	bexarotene [16, 21, 141]	bexarotene [16, 21, 141]	bexarotene [16, 21, 141]
Selective agonists	CD3254 [48]	–	–

### Further reading on 2B. Retinoid X receptors

Germain P et al. (2006) International Union of Pharmacology. LXIII. Retinoid X receptors. Pharmacol. Rev. 58: 760‐72 [PMID:17132853]

Long MD et al. (2015) Vitamin D receptor and RXR in the post‐genomic era. J Cell Physiol 230: 758‐66 [PMID:25335912]

Menendez‐Gutierrez MP et al. (2017) The multi‐faceted role of retinoid X receptor in bone remodeling. Cell Mol Life Sci 74: 2135‐2149 [PMID:28105491]

## 2C. Testicular receptors

### Overview

Testicular receptors (nomenclature as agreed by the NC‐IUPHAR Subcommittee on Nuclear Hormone Receptors [6]) have yet to be officially paired with an endogenous ligand, although testicular receptor 4 has been reported to respond to retinoids.

**Table:** 

Nomenclature	Testicular receptor 2	Testicular receptor 4
Systematic nomenclature	NR2C1	NR2C2
HGNC, UniProt	NR2C1, P13056	NR2C2, P49116
Endogenous agonists	–	retinol [169], tretinoin [169]
Comments	Forms a heterodimer with TR4; gene disruption appears without effect on testicular development or function [132].	Forms a heterodimer with TR2.

### Further reading on 2C. Testicular receptors

Benoit G et al. (2006) International Union of Pharmacology. LXVI. Orphan nuclear receptors. Pharmacol. Rev. 58: 798‐836 [PMID:17132856]

Dahlman‐Wright K et al. (2006) Overview of nomenclature of nuclear receptors. Pharmacol Rev 58: 685‐704 [PMID:17132848]

Safe S et al. (2014) Minireview: role of orphan nuclear receptors in cancer and potential as drug targets. Mol Endocrinol 28: 157‐72 [PMID:24295738]

Wu D et al. (2016) The emerging roles of orphan nuclear receptors in prostate cancer. Biochim. Biophys. Acta 1866: 23‐36 [PMID:27264242]

## 2E. Tailless‐like receptors

### Overview

Tailless‐like receptors (nomenclature as agreed by the NC‐IUPHAR Subcommittee on Nuclear Hormone Receptors [6]) have yet to be officially paired with an endogenous ligand.

**Table:** 

Nomenclature	TLX	PNR
Systematic nomenclature	NR2E1	NR2E3
HGNC, UniProt	NR2E1, Q9Y466	NR2E3, Q9Y5X4
Comments	Gene disruption is associated with abnormal brain development [75, 104].	–

### Further reading on 2E. Tailless‐like receptors

Benod C et al. (2016) TLX: An elusive receptor. J. Steroid Biochem. Mol. Biol. 157: 41‐7 [PMID:26554934]

Benoit G et al. (2006) International Union of Pharmacology. LXVI. Orphan nuclear receptors. Pharmacol. Rev. 58: 798‐836 [PMID:17132856]

Germain P et al. (2006) Overview of nomenclature of nuclear receptors. Pharmacol. Rev. 58: 685‐704 [PMID:17132848]

## 2F. COUP‐TF‐like receptors

### Overview

COUP‐TF‐like receptors (nomenclature as agreed by the NC‐IUPHAR Subcommittee on Nuclear Hormone Receptors [6]) have yet to be officially paired with an endogenous ligand.

**Table:** 

Nomenclature	COUP‐TF1	COUP‐TF2	V‐erbA‐related gene
Systematic nomenclature	NR2F1	NR2F2	NR2F6
HGNC, UniProt	NR2F1, P10589	NR2F2, P24468	NR2F6, P10588
Comments	Gene disruption is perinatally lethal [120].	Gene disruption is embryonically lethal [117].	Gene disruption impairs CNS development [151].

### Further reading on 2F. COUP‐TF‐like receptors

Benoit G et al. (2006) International Union of Pharmacology. LXVI. Orphan nuclear receptors. Pharmacol. Rev. 58: 798‐836 [PMID:17132856]

Germain P et al. (2006) Overview of nomenclature of nuclear receptors. Pharmacol. Rev. 58: 685‐704 [PMID:17132848]

Wu D et al. (2016) The emerging roles of orphan nuclear receptors in prostate cancer. Biochim. Biophys. Acta 1866: 23‐36 [PMID:27264242]

Wu SP et al. (2016) Choose your destiny: Make a cell fate decision with COUP‐TFII. J Steroid Biochem Mol Biol 157: 7‐12 [PMID:26658017]

## 3B. Estrogen‐related receptors

### Overview

Estrogen‐related receptors (nomenclature as agreed by the NC‐IUPHAR Subcommittee on Nuclear Hormone Receptors [6]) have yet to be officially paired with an endogenous ligand.

**Table:** 

Nomenclature	Estrogen‐related receptor‐*α*	Estrogen‐related receptor‐*β*	Estrogen‐related receptor‐*γ*
Systematic nomenclature	NR3B1	NR3B2	NR3B3
HGNC, UniProt	ESRRA, P11474	ESRRB, O95718	ESRRG, P62508
Comments	Activated by some dietary flavonoids [138]; activated by the synthetic agonist GSK4716 [181] and blocked by XCT790 [156].	May be activated by DY131 [162].	May be activated by DY131 [162].

### Further reading on 3B. Estrogen‐related receptors

Benoit G et al. (2006) International Union of Pharmacology. LXVI. Orphan nuclear receptors. Pharmacol. Rev. 58: 798‐836 [PMID:17132856]

Divekar SD et al. (2016) Estrogen‐related receptor *β*(ERR*β*) ‐ renaissance receptor or receptor renaissance? Nucl Recept Signal 14: e002 [PMID:27507929]

Germain P et al. (2006) Overview of nomenclature of nuclear receptors. Pharmacol. Rev. 58: 685‐704 [PMID:17132848]

Tam IS et al. (2016) There and back again: The journey of the estrogen‐related receptors in the cancer realm. J Steroid Biochem Mol Biol 157: 13‐9[PMID:26151739]

Wu D et al. (2016) The emerging roles of orphan nuclear receptors in prostate cancer. Biochim. Biophys. Acta 1866: 23‐36 [PMID:27264242]

## 4A. Nerve growth factor IB‐like receptors

### Overview

Nerve growth factor IB‐like receptors (nomenclature as agreed by the NC‐IUPHAR Subcommittee on Nuclear Hormone Receptors [6]) have yet to be officially paired with an endogenous ligand.

**Table:** 

Nomenclature	Nerve Growth factor IB	Nuclear receptor related 1	Neuron‐derived orphan receptor 1
Systematic nomenclature	NR4A1	NR4A2	NR4A3
HGNC, UniProt	NR4A1, P22736	NR4A2, P43354	NR4A3, Q92570
Comments	An endogenous agonist, cytosporone B, has been described [164], although structural analysis and molecular modelling has not identified a ligand binding site [4, 40, 150].	–	–

### Further reading on 4A. Nerve growth factor IB‐like receptors

Benoit G et al. (2006) International Union of Pharmacology. LXVI. Orphan nuclear receptors. Pharmacol. Rev. 58: 798‐836 [PMID:17132856]

Germain P et al. (2006) Overview of nomenclature of nuclear receptors. Pharmacol. Rev. 58: 685‐704 [PMID:17132848]

Ranhotra HS. (2015) The NR4A orphan nuclear receptors: mediators in metabolism and diseases. J Recept Signal Transduct Res 35: 184‐8 [PMID:25089663]

Rodriguez‐Calvo R et al. (2017) The NR4A subfamily of nuclear receptors: potential new therapeutic targets for the treatment of inflammatory diseases. Expert Opin Ther Targets 21: 291‐304 [PMID:28055275]

Safe S et al. (2016) Nuclear receptor 4A (NR4A) family ‐ orphans no more. J Steroid Biochem Mol Biol 157: 48‐60 [PMID:25917081]

## 5A. Fushi tarazu F1‐like receptors

### Overview

Fushi tarazu F1‐like receptors (nomenclature as agreed by the NC‐IUPHAR Subcommittee on Nuclear Hormone Receptors [6]) have yet to be officially paired with an endogenous ligand.

**Table:** 

Nomenclature	Steroidogenic factor 1	Liver receptor homolog‐1
Systematic nomenclature	NR5A1	NR5A2
HGNC, UniProt	NR5A1, Q13285	NR5A2, O00482
Comments	Reported to be inhibited by AC45594 [32] and SID7969543 [90].	–

### Further reading on 5A. Fushi tarazu F1‐like receptors

Benoit G et al. (2006) International Union of Pharmacology. LXVI. Orphan nuclear receptors. Pharmacol. Rev. 58: 798‐836 [PMID:17132856]

Garattini E et al. (2016) Lipid‐sensors, enigmatic‐orphan and orphan nuclear receptors as therapeutic targets in breast‐cancer. Oncotarget. 7: 42661‐42682 [PMID:26894976]

Germain P et al. (2006) Overview of nomenclature of nuclear receptors. Pharmacol. Rev. 58: 685‐704 [PMID:17132848]

Zhi X et al. (2016) Structures and regulation of non‐X orphan nuclear receptors: A retinoid hypothesis. J Steroid Biochem Mol Biol. 157: 27‐40 [PMID:26159912]

Zimmer V et al. (2015) Nuclear receptor variants in liver disease. Dig Dis. 33: 415‐9 [PMID:26045277]

## 6A. Germ cell nuclear factor receptors

### Overview

Germ cell nuclear factor receptors (nomenclature as agreed by the NC‐IUPHAR Subcommittee on Nuclear Hormone Receptors [6]) have yet to be officially paired with an endogenous ligand.

**Table:** 

Nomenclature	Germ cell nuclear factor
Systematic nomenclature	NR6A1
HGNC, UniProt	NR6A1, Q15406

### Further reading on 6A. Germ cell nuclear factor receptors

Benoit G et al. (2006) International Union of Pharmacology. LXVI. Orphan nuclear receptors. Pharmacol. Rev. 58: 798‐836 [PMID:17132856]

Garattini E et al. (2006) Lipid‐sensors, enigmatic‐orphan and orphan nuclear receptors as therapeutic targets in breast‐cancer. Oncotarget. 7: 42661‐42682 [PMID:26894976]

Germain P et al. (2006) Overview of nomenclature of nuclear receptors. Pharmacol. Rev. 58: 685‐704 [PMID:17132848]

Safe S et al. ((2014) Minireview: role of orphan nuclear receptors in cancer and potential as drug targets. Mol Endocrinol. 28: 157‐72 [PMID:24295738]

Zhi X et al. (2016) Structures and regulation of non‐X orphan nuclear receptors: A retinoid hypothesis. J Steroid Biochem Mol Biol. 157: 27‐40 [PMID:26159912]

## 0B. DAX‐like receptors

### Overview

Dax‐like receptors (nomenclature as agreed by the NC‐IUPHAR Subcommittee on Nuclear Hormone Receptors [6]) have yet to be officially paired with an endogenous ligand.

**Table:** 

Nomenclature	DAX1	SHP
Systematic nomenclature	NR0B1	NR0B2
HGNC, UniProt	NR0B1, P51843	NR0B2, Q15466

### Further reading on 0B. DAX‐like receptors

Benoit G et al. (2006) International Union of Pharmacology. LXVI. Orphan nuclear receptors. Pharmacol. Rev. 58: 798‐836 [PMID:17132856]

Garattini E et al. (2016) Lipid‐sensors, enigmatic‐orphan and orphan nuclear receptors as therapeutic targets in breast‐cancer. Oncotarget 7: 42661‐42682 [PMID:26894976]

Germain P et al. (2006) Overview of nomenclature of nuclear receptors. Pharmacol. Rev. 58: 685‐704 [PMID:17132848]

Safe S et al. (2014) Minireview: role of orphan nuclear receptors in cancer and potential as drug targets. Mol Endocrinol 28: 157‐72 [PMID:24295738]

Wu D et al. (2016) The emerging roles of orphan nuclear receptors in prostate cancer. Biochim. Biophys. Acta 1866: 23‐36 [PMID:27264242]

## Steroid hormone receptors

### Overview

Steroid hormone receptors (nomenclature as agreed by the NC‐IUPHAR Subcommittee on Nuclear Hormone Receptors [30, 87]) are nuclear hormone receptors of the NR3 class, with endogenous agonists that may be divided into 3‐hydroxysteroids (estrone and 17*β*‐estradiol) and 3‐ketosteroids (dihydrotestosterone[DHT], aldosterone, cortisol, corticosterone, progesterone and testosterone). These receptors exist as dimers coupled with chaperone molecules (such as hsp90*β*(HSP90AB1, P08238) and immunophilin FKBP52:FKBP4, Q02790), which are shed on binding the steroid hormone. Although rapid signalling phenomena are observed [83, 119], the principal signalling cascade appears to involve binding of the activated receptors to nuclear hormone response elements of the genome, with a 15‐nucleotide consensus sequence AGAACAnnnTGTTCT (i.e. an inverted palindrome) as homo‐ or heterodimers. They also affect transcription by protein‐protein interactions with other transcription factors, such as activator protein 1 (AP‐1) and nuclear factor *κ*B (NF‐*κ*B). Splice variants of each of these receptors can form functional or non‐functional monomers that can dimerize to form functional or non‐functional receptors. For example, alternative splicing of PR mRNA produces A and B monomers that combine to produce functional AA, AB and BB receptors with distinct characteristics [145].

A 7TM receptor responsive to estrogen (GPER1, Q99527, also known as GPR30, see [118]) has been described. Human orthologues of 7TM 'membrane progestin receptors' (PAQR7, PAQR8 and PAQR5), initially discovered in fish [170, 171], appear to localize to intracellular membranes and respond to 'non‐genomic' progesterone analogues independently of G proteins [134].

## 3A. Estrogen receptors

### Overview

Estrogen receptor (ER) activity regulates diverse physiological processes via transcriptional modulation of target genes. The selection of target genes and the magnitude of the response, be it induction or repression, are determined by many factors, including the effect of the hormone ligand and DNA binding on ER structural conformation, and the local cellular regulatory environment. The cellular environment defines the specific complement of DNA enhancer and promoter elements present and the availability of coregulators to form functional transcription complexes. Together, these determinants control the resulting biological response.

**Table:** 

Nomenclature	Estrogen receptor‐*α*	Estrogen receptor‐*β*
Systematic nomenclature	NR3A1	NR3A2
HGNC, UniProt	ESR1, P03372	ESR2, Q92731
Endogenous agonists	estriol [74], estrone [74]	–
Selective agonists	propylpyrazoletriol [73, 135], ethinylestradiol [62]	WAY200070 [93], diarylpropionitrile [100, 135], prinaberel [29, 93]
Sub/family‐selective antagonists	bazedoxifene (pIC_50_ 7.6) [103]	bazedoxifene (pIC_50_ 7.1) [103]
Selective antagonists	clomiphene (p*K*_i_ 8.9) [3], methyl‐piperidino‐pyrazole (p*K*_i_ 8.6) [139]	R,R‐THC (p*K*_i_ 8.4) [99, 140], PHTPP (p*K*_i_ 6.9) [168]

### Comments

R,R‐THC exhibits partial agonist activity at ER*α*[99, 140]. Estrogen receptors may be blocked non‐selectively by tamoxifen and raloxifene and labelled by [^3^H]17*β*‐estradiol and [^3^H]tamoxifen. Many agents thought initially to be antagonists at estrogen receptors appear to have tissue‐specific efficacy (e.g. Tamoxifen is an antagonist at estrogen receptors in the breast, but is an agonist at estrogen receptors in the uterus), hence the descriptor SERM (selective estrogen receptor modulator) or SnuRM (selective nuclear receptor modulator). Y134 has been suggested to be an ER*α*‐selective estrogen receptor modulator [111].

### Further reading on 3A. Estrogen receptors

Dahlman‐Wright K et al. (2016) Estrogen Receptor Ligands: A Review (2013‐2015). Sci Pharm 84: 409‐427 [PMID:28117309]

Gonzalez‐Sanchez E et al. (2015) Nuclear receptors in acute and chronic cholestasis. Dig Dis 33: 357‐66 [PMID:26045270]

Gustafsson (2006) International Union of Pharmacology. LXIV. Estrogen receptors. Pharmacol Rev 58: 773‐81 [PMID:17132854]

Hewitt SC et al. (2016) What's new in estrogen receptor action in the female reproductive tract. J. Mol. Endocrinol. 56: R55‐71 [PMID:26826253]

Jameera Begam A et al. (2017) Estrogen receptor agonists/antagonists in breast cancer therapy: A critical review. Bioorg Chem 71: 257‐274 [PMID:28274582]

Warner M et al. (2017) Estrogen Receptor *β* as a Pharmaceutical Target. Trends Pharmacol. Sci. 38: 92‐99 [PMID:27979317]

## 3C. 3‐Ketosteroid receptors

**Table:** 

Nomenclature	Androgen receptor	Glucocorticoid receptor
Systematic nomenclature	NR3C4	NR3C1
HGNC, UniProt	AR, P10275	NR3C1, P04150
Rank order of potency	dihydrotestosterone [142]>testosterone	cortisol, corticosterone≫aldosterone, deoxycortisone [125]
Selective agonists	testosterone propionate [95], mibolerone [49], fluoxymesterone [61], methyltrienolone [148], dromostanolone propionate	fluticasone propionate [10], beclometasone [3], methylprednisolone [3], fluocinonide [3], betamethasone [3], budesonide [102]
Selective antagonists	bicalutamide (p*K*_i_ 7.7) [70], PF0998425 (pIC_50_ 7.1–7.5) [85], enzalutamide (pIC_50_ 7.4) [143], nilutamide (pIC_50_ 7.1–7.1) [133], hydroxyflutamide (pEC_50_ 6.6) [148], galeterone (pIC_50_ 6.4) [56], flutamide (p*K*_i_ 5.4) [147]	onapristone (pIC_50_ 7.6) [165], ZK112993
Labelled ligands	[^3^H]dihydrotestosterone (Selective Agonist), [^3^H]methyltrienolone (Selective Agonist), [^3^H]mibolerone (Agonist)	[^3^H]dexamethasone (Agonist)

**Table:** 

Nomenclature	Mineralocorticoid receptor	Progesterone receptor
Systematic nomenclature	NR3C2	NR3C3
HGNC, UniProt	NR3C2, P08235	PGR, P06401
Rank order of potency	corticosterone, cortisol, aldosterone [58, 125], progesterone [125]	progesterone [38]
Selective agonists	–	medroxyprogesterone (Affinity at human PR‐A) [166], ORG2058, levonorgestrel [9, 128]
Selective antagonists	finerenone (pIC_50_ 7.7) [20], eplerenone (p*K*_i_ 6.9) [5], onapristone (pIC_50_ 6.3) [165], RU28318, ZK112993	ulipristal acetate (pIC_50_ 9.7) [123], mifepristone (Mixed) (p*K*_i_ 9) [167], onapristone (p*K*_i_ 7.7) [54], ZK112993
Labelled ligands	[^3^H]aldosterone (Selective Agonist) [44, 137] – Rat	[^3^H]ORG2058 (Selective Agonist)

### Comments

[^3^H]dexamethasone also binds to MR in vitro. PR antagonists have been suggested to subdivide into Type I (e.g. onapristone) and Type II (e.g. ZK112993) groups. These groups appear to promote binding of PR to DNA with different efficacies and evoke distinct conformational changes in the receptor, leading to a transcription‐neutral complex [43, 82]. Mutations in AR underlie testicular feminization and androgen insensitivity syndromes, spinal and bulbar muscular atrophy (Kennedy's disease).

### Further reading on 3C. 3‐Ketosteroid receptors

Baker ME et al. (2017) 30 YEARS OF THE MINERALOCORTICOID RECEPTOR: Evolution of the mineralocorticoid receptor: sequence, structure and function. J Endocrinol 234: T1‐T16 [PMID:28468932]

Carroll JS et al. (2017) Deciphering the divergent roles of progestogens in breast cancer. Nat Rev Cancer 17: 54‐64 [PMID:27885264]

Cohen DM et al. (2017) Nuclear Receptor Function through Genomics: Lessons from the Glucocorticoid Receptor. Trends Endocrinol Metab 28: 531‐540 [PMID:28495406]

de Kloet ER et al. (2017) Brain mineralocorticoid receptor function in control of salt balance and stress‐adaptation. Physiol. Behav. [PMID:28089704]

Garg D, et al. (2017) Progesterone‐Mediated Non‐Classical Signaling. Trends Endocrinol Metab [PMID:28651856]

Lu NZ et al. (2006) International Union of Pharmacology. LXV. The pharmacology and classification of the nuclear receptor superfamily: glucocorticoid, mineralocorticoid, progesterone, and androgen receptors. Pharmacol Rev 58: 782‐97 [PMID:17132855]

Lucas‐Herald AK et al. (2017) Genomic and non‐genomic effects of androgens in the cardiovascular system: clinical implications. Clin Sci (Lond) 131: 1405‐1418 [PMID:28645930]

Wadosky KM et al. (2017) Androgen receptor splice variants and prostate cancer: From bench to bedside. Oncotarget 8: 18550‐18576 [PMID:28077788]

Weikum ER et al. (2017) Glucocorticoid receptor control of transcription: precision and plasticity via allostery. Nat Rev Mol Cell Biol 18: 159‐174 [PMID:28053348]
